# How is patient trust transferred from online medical platforms to offline?

**DOI:** 10.3389/fpubh.2025.1535218

**Published:** 2025-02-28

**Authors:** Lin Liang

**Affiliations:** ^1^Business School, Central South University, Changsha, China; ^2^Xiangya Hospital, Central South University, Changsha, China

**Keywords:** online healthcare, patient online trust, patient offline trust, reputation mechanism, trust transfer

## Abstract

**Introduction:**

The function of the internet medical platform has expanded from online consultation to offline diagnosis and treatment appointment, forming a complete service process combining online and offline, improving the patient's medical experience and promoting the transfer of online trust to offline. However, the existing studies pay insufficient attention to the dynamic and multi-stage characteristics of online medical trust, especially the lack of in-depth discussion on the trust transfer of patients from online to offline.

**Methods:**

This study builds a patient trust transfer model based on relevant theories, and analyzes the influence mechanism of online reputation feedback on patients' online and offline trust combined with text mining technologies such as sentiment analysis. The research adopts the multi-dimensional analysis method, comprehensively considers the online and offline scenarios, and reveals the key drivers of trust transfer through large-scale data analysis.

**Results:**

The study found that doctors' online reputation feedback and interaction quality were important factors affecting patients' trust transfer. Positive online interaction and high-quality reputation feedback significantly enhanced patient trust and promoted the transfer of online trust to offline. The trust transfer process is dynamic and multi-stage, and the influencing factors of different stages are different. The study also revealed the significant difference in trust mechanism between online medicine and traditional medicine.

**Discussion:**

This study revealed the formation and transfer mechanism of trust in online health care by building a trust transfer model, filling the gap in related research. The results provide practical guidance for the online medical platform to optimize the service process and enhance the trust of patients. In the future, we can further explore the trust transfer mechanism under different cultural backgrounds to promote the globalization of Internet medicine.

## 1 Introduction

Online healthcare, as a new form of medical consultation in modern society, has won the favor of a large number of users due to its convenience and efficiency since its inception, and has maintained a stable growth momentum (1). Online medical platforms provide various communication methods such as text, images, and videos, making communication between doctors and patients more comprehensive and convenient. Meanwhile, doctors can utilize fragmented time for online diagnosis and treatment, improving work efficiency and providing patients with more medical options (2). In other words, online healthcare has improved the accuracy and personalization of medical services through technologies such as big data and artificial intelligence, thereby enhancing the quality of medical services.

At the same time, online medical platforms have broken the limitations of geography and time through digital means, enabling patients to access medical services more conveniently. On the one hand, it has increased the coverage of medical services. Online medical platforms enable patients in remote areas or with limited mobility to access high-quality medical resources, improving the coverage of medical services. On the other hand, it reduces the waiting time for patients. Patients do not need to go to the hospital in person, they can obtain doctors' consultation and advice through online platforms, thereby reducing waiting time for treatment (3).

In addition, online medical platforms play an important role in reducing patient medical expenses and improving medical efficiency (2). One is to reduce medical expenses. Online medical platforms reduce additional costs for patients, such as transportation and accommodation, by providing remote consultation, online diagnosis and treatment services. Secondly, it has improved medical efficiency. Digital medical methods enable doctors to have a more accurate understanding of patients' conditions, thereby developing more precise treatment plans, reducing unnecessary examinations and drug use, and lowering medical costs. Thirdly, some platforms also provide financial services such as medical insurance and installment payments, further enhancing patients' medical experience.

However, we also need to be aware that the development of online medical platforms still faces many challenges and problems. For example, aspects such as medical quality and safety, doctor-patient trust, technology and privacy protection need to be further improved and optimized.

In summary, as an important component of the modern healthcare system, online medical platforms provide patients with convenient and efficient medical services, while also offering doctors a more flexible way of working. However, the issue of doctor-patient trust has always been one of the key challenges in the development of online healthcare platforms. One is the issue of data security and privacy protection. Despite the various data security measures taken by online medical platforms, once a data breach occurs, it will cause huge harm to patients and seriously damage the reputation of medical institutions. In addition, some platforms have shortcomings in privacy protection, such as collecting, using, or leaking personal information without the patient's consent, leading to a decrease in patient trust. Secondly, the quality of service varies greatly. Due to differences in internal management and personnel quality within medical institutions, some patients may encounter situations such as indifferent service attitudes and non-standard diagnosis and treatment during the medical process, leading to a breakdown of trust. The third issue is poor communication. In the actual diagnosis and treatment process, due to unequal information and insufficient communication skills between doctors and patients, patients often find it difficult to fully understand their condition and treatment plan, resulting in doubts and concerns, which affects the establishment of trust between doctors and patients (4). Therefore, to truly establish a stable doctor-patient trust relationship, it requires joint efforts from various aspects such as the platform, doctors, patients, and the government.

Moreover, in the process of providing medical services on online medical platforms, due to spatial barriers and the particularity of the medical industry, the reputation feedback mechanism of the platform has become a key factor in establishing trust between users and doctors (5). The solution to this problem plays a crucial role in enhancing the trust of online medical platforms among users and promoting the further development of online healthcare. Although online healthcare has experienced rapid development in the context of online consumption, its characteristics differ significantly from online consumption. Online healthcare mainly provides professional services rather than property transactions, which makes users have higher expectations for doctors' professional abilities and service quality. Meanwhile, the high-frequency real-time user demands driven by mobile technology also make trust building and risk perception in online medical processes more complex and variable (6).

With the continuous expansion of medical and health platform and website functions, online consultation and offline diagnosis and treatment appointment have formed a complete Internet medical service process. When patients consult online, they can have in-depth communication with doctors to understand their condition and treatment plan; when offline medical treatment is needed, it can be easily scheduled through the platform, achieving seamless integration between online consultation and offline medical treatment (7). This process not only greatly improves the patient's medical experience, but also promotes the transfer of patient trust from online to offline. In the environment provided by online medical platforms, real-time interaction between patients and doctors has become possible. Patients can understand the service quality or personal characteristics of doctors through the platform reputation feedback mechanism, and these signals serve as important clues that will directly affect the formation of patient trust (8). Therefore, how to better utilize these signals and improve the trust of online medical platforms has become an important issue that urgently needs to be addressed.

By systematically reviewing the historical evolution of doctor-patient trust and the current research status of doctor-patient trust by domestic and foreign scholars, we can clearly see that online healthcare, as an innovative model that integrates information technology and medical services, is gradually changing the traditional medical landscape (9–12). From the initial exploration of technological applications to the current in-depth attention to the needs of users and patients, research in the field of online healthcare is constantly expanding and deepening. However, although scholars have begun to pay attention to issues such as online health platforms and online health information, there is still insufficient attention to the core issue of doctor-patient trust, especially the transfer of trust between patients on online medical platforms from online to offline.

Current research focuses more on the antecedents and consequences of trust in specific scenarios, neglecting the dynamic and multi-stage nature of trust. Trust is complex and multidimensional, evolving over time and space, and requires a deeper exploration of its evolutionary patterns and influencing factors beyond a single stage analysis (13). As a new medical service model, online healthcare differs from traditional healthcare in terms of doctor-patient interaction and trust mechanisms. It has characteristics such as anonymity, remote access, and immediacy, and requires specialized research on its trust evolution mechanism (2). The trust issue in online healthcare involves temporal and spatial transformation. Trust may be established online first, and then consolidated and improved through offline medical services, requiring a comprehensive consideration of various factors both online and offline. Therefore, a comprehensive and systematic research method is needed, including multidimensional considerations and comprehensive consideration of online and offline scenarios, to accurately grasp the essence and laws of doctor-patient trust, optimize doctor-patient relationships, improve the quality of medical services, and help understand and solve the crisis of doctor-patient trust both online and offline.

## 2 Theory

### 2.1 Online healthcare

#### 2.1.1 Concept of online healthcare

Internet medicine is the product of the combination of network communication technology and the field of medical and health care. Institutions and personnel with medical qualifications provide medical and health services through electronic communication technology, computers, mobile terminals and other information tools. This field developed early in foreign countries. The U.S. government started research on “telemedicine” related policies in 1976 and first proposed the concept of “telemedicine” the following year. In the 1990s, other developed countries and regions also actively participated in the construction of regional medical and health informatization. For example, the European Union launched the “European e-health action plan” in 2004, while the UK launched the national health informatization project (npfit) in 2002. Since then, smart phones, PDAs and other mobile terminals have been widely used in the medical field, which not only helps the efficient collection and management of hospital data, but also greatly improves the convenience of users to obtain medical services and significantly optimizes the efficiency of medical services (14).

#### 2.1.2 Difference between online healthcare and traditional healthcare

There are many differences between online health care and traditional health care systems. These differences are mainly reflected in medical channels, diagnosis and treatment methods, data storage and management, medical services and expenses (15).

First, access to medical treatment. Online health care uses the Internet as the medium to realize the storage, transmission, communication and support of medical services through digital and information technology. Patients only need to register, consult and pay on their mobile phones to obtain diagnosis, treatment and consulting services, eliminating the tedious process of queuing, waiting and registration. Traditional medical treatment is mainly carried out through offline medical institutions such as hospitals, clinics and community health service centers. Patients need to fill in medical records, queue up, register, and then accept the diagnosis and treatment of doctors. The process is relatively cumbersome and may need to wait for a long time. The second is the diagnosis and treatment method. Patients in the online medical platform can communicate with doctors through online video, graphics and other forms. This “cloud medical” method greatly reduces the problems of difficult and expensive medical treatment, and can also solve the situation that some patients cannot go to the hospital at home. Traditional medicine relies on professional equipment and doctors' examination, auxiliary diagnosis and treatment methods, such as electrocardiogram, B-ultrasound, surgery, etc. Third, medical services. Online medicine can provide round the clock online consultation and diagnosis services, as well as online prescription, drug purchase and distribution services. Online consultation is no longer limited by time and space, extending the time span of medical services. Traditional medicine usually needs to be treated at a fixed time and place. Although the diagnosis and treatment experience and academic accumulation are well-known, the service flexibility and convenience are relatively weak. Fourth, expenses. Online medical costs are relatively low, because there is no need to rely on large medical institutions, nor need to pay the human costs of hospitals and other institutions. The cost of traditional medical treatment is relatively high, especially when it comes to high-end examination and treatment. Fifth, risk management. There are great differences in the quality of medical students on the online medical platform. Some doctors may not have a practicing doctor certificate or have a low level of knowledge, causing risks to the treatment of patients. At the same time, the risk of personal privacy disclosure is high, because patients need to input personal information into the Internet system. There is a risk of misdiagnosis, because doctors cannot diagnose patients face-to-face, nor can they use actual equipment for examination. Although traditional medicine also has the risk of misdiagnosis, it is relatively low, because doctors can diagnose and treat face-to-face. The risk of data leakage is relatively low, because the traditional medical data storage and management methods are relatively safe.

To sum up, online health care and traditional health care systems have their own advantages and disadvantages. How to effectively combine the two to form a multi-party coexisting health care system is an urgent problem for us to solve.

#### 2.1.3 Current status of online medical research

At present, the related research of Internet medicine mainly focuses on patients (users), and the research topics mainly cover three aspects. One is user health information behavior in the Internet environment. Study how users collect, evaluate and use health information. Users mainly obtain the information they need through health information search, and will evaluate the quality of the information, which is affected by factors such as information sources, communication channels, etc. Ultimately, users will make decisions based on information (16). The second is the impact of online medical and health communities and social media on health. Online medical and health communities not only provide health information, but also provide social support, including information support, emotional support and companionship. Social media also enriches users' sources of medical and health information and provides users with a platform to express their health concepts. These platforms help users form a mutually supportive patient friend network and improve health (17). Third, health privacy and trust research. Users are worried about the privacy protection of personal health information, which will affect their acceptance of online medical and health services. Privacy concerns will negatively affect user trust, and then affect the adoption of mobile health care. Strengthening privacy protection and security regulations can alleviate this negative effect, and also help build trust in online health information interaction (18).

### 2.2 Trust theory

#### 2.2.1 Concept of trust theory

Trust, as a multidimensional and interdisciplinary concept, has presented different research perspectives and definitions in various fields. In the field of social psychology, trust is regarded as a comprehensive reflection of individual psychology, personality traits, and behavioral performance. It focuses on the trust relationship between people and its impact on individual behavior. Scholars in this field, such as Deutsch, have revealed the central role of trust in interpersonal relationships by exploring the role of trust in conflict resolution (19). Rotter and Julian have also provided many explanations regarding trust between individuals and organizations, among which the expectation given to a particular individual or organization is what we call trust (20). Brahm and Kunze proposed that trust is a feeling, expectation, or belief of a person (21).

Management regards trust as an important mechanism within an organization, which helps to reduce uncertainty and risk and improve management performance. Driscoll believes that trust is the belief of decision makers that implementing a certain behavior will result in a beneficial outcome for themselves (22). Mayer et al.'s (23) study emphasizes the important role of trust in decision-making, cost reduction, and relationship regulation. Hwang et al. proposed that trust is conducive to promoting the formation and stability of cooperative relationships between individuals and between individuals and organizations (24).

#### 2.2.2 Research on trust transfer

Doney and Cannon proposed and explained trust transfer, which refers to the transfer of trusted things or people to unknown people or things, thus forming certain special relationships (25). The foundation of trust is an important source of trust, but it also requires the joint action of other factors to truly generate trust. Many factors, such as the environment in which the trust target is located, can affect the trust subject's perception and evaluation of the target (26). Trust transfer is an important component of the trust formation process. Xu found through research that online comments attract tourists' attention, which is influenced by both positive and negative comments. Among them, positive comments promote the establishment and strengthening of user trust. According to relevant research, trust objects can be transferred, and these transfers are all influenced by the source of trust (27). For example, Flaherty pointed out in her research that when a trusted third party proves that a stranger is trustworthy, they largely decide to trust the stranger (28). Plavini summarized the impact of online trust in retail enterprises based on the theory of trust antecedents, and found that there is a significant positive relationship between retail performance and online trust level, and product type also affects the trust relationship (29). In the online environment, people often rely on the experiences of others, such as online comments, to reduce their doubts and uncertainties, thereby establishing trust in unfamiliar targets. This is the process of trust transmission (30).

There are various psychological processes involved in building trust, and many analyses have been conducted based on trust transmission models. For example, foreign scholars Doney and Cannon proposed a trust process model, which is divided into five processes: computation, prediction, ability, intention, and transmission (25). Among them, transmission refers to the evaluation and judgment of trust in organizations or individuals. The organization or individual can assign trustworthy characteristics to the authenticated person. This also indicates that when consumers have little or no knowledge of the trust object used as a basis for judgment, trust can be transferred from high trust sources to the target object, such as third-party networks, authentication and evaluation by trust assessment agencies, etc. They will transfer trust to the corresponding online merchants. Scholars represented by McKnight have identified three methods for building trust from the perspectives of institutions, knowledge transfer, and trust transfer. Firstly, trust can be transmitted in different processes (31). For example, the trust of the principal during the communication process may be directly affected by the third party or target party involved in the trust transfer (32). Yang et al. (33) found through empirical research that users' trust in suppliers' offline stores can be transferred to their online stores. Zhao et al. provided a detailed summary of the implementation of trust transfer and proposed a trust transfer mechanism, pointing out that trust transfer cannot be separated from the scope of trust. They also analyzed two types of trust: functional trust and recommendation trust (34).

#### 2.2.3 Current status of trust theory research

Trust plays an important role in the medical and health system, and the medical services provided by doctors are regarded as a unique trust commodity. With the rise of online medical services, scholars at home and abroad began to explore the trust problem in this new situation. At present, the research on trust in online medical context is mainly carried out in three areas: online medical and health information, online medical and health websites, and mobile medicine. Its core is to analyze the influencing factors of trust and its results.

The Internet has become the main way for people to obtain health information. However, the medical and health information on the Internet is rich and complex, and it is easy to be misled by wrong information for users who lack medical knowledge. Therefore, it is particularly important to build a trust relationship between doctors and patients, which can not only help users screen information more effectively, but also further promote the harmony of doctor-patient relationship. The empirical study of Harris et al. revealed multiple influencing factors of online trust, including the quality and neutrality of online health information, as well as users' perceived threats and new confirmations. These factors have a significant positive impact on users' online trust, and then affect their willingness to accept suggestions (35).

Online medical and health websites provide users with diverse medical and health services. In this context, scholars have conducted in-depth research on trust. Ko et al. divided trust into cognitive trust and emotional trust, and constructed the corresponding trust model to explore the establishment mechanism of user trust in online medical communities (36). Seckler et al.'s research found that user interface, demand satisfaction, responsiveness, security and other factors have a significant impact on user trust. At the same time, trust will further affect user satisfaction and loyalty (37).

In addition, some scholars have also explored the issue of trust in the field of mobile medicine. Guo et al. (38) analyzed the trust and adoption of mHealth services by users at different ages from the perspective of privacy and personalization. Kesharwani et al. (39), combined with the technology acceptance model, explored the influence of trust, risk, ease of use and usefulness on the use of mobile health applications by AIDS patients. Akter et al. (40), taking low-income people as the research object, constructed a model of continuous use of mobile health care, and found that user trust has a significant impact on their willingness to continue to use.

### 2.3 Reputation mechanism

The reputation mechanism, as an indispensable part of social communication, carries different meanings and values in different fields. In the field of online markets, the definition of reputation is more specific and important: it represents the conditional probability of a person acting in a specific way and becomes one of the key factors for the success of online transactions or services (41). The online reputation feedback mechanism has just taken advantage of the two-way communication characteristics of the Internet and the huge amount of information to establish a reputation information network dedicated to providing communication and feedback for online trading parties (42). This mechanism effectively prevents the occurrence of moral hazard and mitigates the potential harm caused by adverse selection by designing clever reward and punishment mechanisms and information learning mechanisms, thereby greatly improving the efficiency and security of online transactions (43). In multiple fields, online reputation feedback mechanisms have played a crucial role. In the field of e-commerce, reputation feedback mechanism has become the core of website management. It not only ensures the stability of online transactions and effectively prevents online fraud, but also establishes a solid trust relationship between buyers and sellers, greatly improving market efficiency (42). In the medical field, with the rise of online medical services, the online reputation feedback mechanism has also become an important reference for patients to obtain doctor service information and make reasonable medical decisions (44).

In traditional offline environments, reputation measurement is often difficult due to the difficulty of obtaining and disseminating information. But with the emergence of various social platforms and new media, measuring online reputation has become particularly important (45). Online reputation facilitates communication and decision-making between users and platforms, creating favorable conditions for cooperation between both parties. In the field of online healthcare, the improvement of doctor-patient trust and information asymmetry issues, as well as the transmission of service quality, largely depend on online reputation (46). Practice has shown that the higher the reputation level, the lower the uncertainty and perceived risk of users (47). In the medical field, the problem of information asymmetry between doctors and patients is particularly prominent, and online medical service platforms exacerbate this issue. However, with the development of the Internet, patients are increasingly inclined to search and understand the reputation of doctors through online platforms. The formation of doctors' online reputation mainly relies on patients' online evaluations, feedback, sharing, and promotion by medical institutions. This information quickly spreads through online platforms and has a profound impact on other patients' medical decisions.

### 2.4 Research review

Through the systematic review of domestic and foreign scholars' research on online health care, trust theory, reputation mechanism, and trust in the context of online health care, we can clearly observe the rapid development trend of online health care and its potential optimization space. At the same time, the core role of doctor-patient trust in optimizing the doctor-patient relationship has been widely recognized (10). However, the current research on doctor-patient trust in online medicine still faces several key issues that need to be further explored.

(1) The development and research status of online medicine shows that the field is undergoing a shift from simple technology application to in-depth attention to the needs of users and patients. However, despite the significant progress made in online health care, scholars have focused more on online health communities and online health information related issues, while the discussion on doctor-patient trust is relatively rare. In fact, reasonable service selection and the improvement of doctor-patient trust are the key elements for the sustainable development of online health care. As an important cornerstone of the stable operation of society, trust has been faced with a crisis of trust in the offline environment, and the complexity and anonymity of the Internet environment exacerbate the difficulty of building doctor-patient trust. Due to the particularity of medical services, people tend to rely more on traditional face-to-face offline medical services, which further highlights the seriousness of the problem of doctor-patient trust in the Internet environment. However, at present, the research on doctor-patient trust at home and abroad is mostly limited to offline environment, and the research on doctor-patient trust in online medical context is obviously insufficient.
(2) When integrating the formation and evolution of doctor-patient trust and trust research in the online medical context, we found that although scholars paid high attention to trust issues in the Internet context, these studies were relatively scattered and lack of systematicness. The existing research focuses more on the antecedents and outcomes of trust in a single stage in different scenarios, and rarely incorporates the dynamic and multi-stage characteristics of trust into the analysis framework. Therefore, at present, the research on doctor-patient trust in online medical environment is still relatively single, and there is a lack of comprehensive and systematic research on the formation and evolution of doctor-patient trust, which limits our in-depth understanding of the construction and evolution of doctor-patient trust in online medical environment.
(3) The lack of trust between doctors and patients and the distrust between doctors and patients are one of the most serious problems in the doctor-patient relationship. Although there have been in-depth studies on the dimensions, system evaluation, influencing factors and results of doctor-patient trust in the traditional medical model, there is still a relative lack of research on the doctor-patient trust in the innovative medical model of online medicine. There are significant differences between the doctor-patient interaction mode in online medicine and the traditional medical model, so the influencing factors, trust dimensions and trust results of doctor-patient trust will also be different. In addition, the evolution process of trust in the online medical environment has become more complex, which not only involves the evolution of different time periods, but also spans online and offline. Therefore, it is of great significance to systematically study the construction and evolution of doctor-patient trust in online medical environment, and to explore the formation and evolution mechanism of doctor-patient trust, in order to promote the healthy development of doctor-patient relationship and improve the quality and efficiency of online medical services.

## 3 Hypothesis

### 3.1 Trust transfer

Trust transfer is a unique and important mechanism in building user trust. When we delve deeper into this mechanism, we will find that it is essentially a complex cognitive process (26). In this process, people often transfer their trust in a familiar target to another relatively unfamiliar target based on some kind of correlation between the targets (48). This transfer is not limited to trust exchange between entities, it also involves the transfer of trust in different contexts. This article focuses on the transfer of trust between channels, specifically transferring trust from one channel or context to another. We have seen many examples of this in e-commerce platforms. For example, the research of Naseri et al. (49) revealed how users' trust in traditional offline banks significantly affects their trust in corresponding online banks. This trust transfer has expanded from offline channels to online channels, proving that even in a digital environment, users' trust in traditional institutions still has strong continuity. Similarly, scholars such as Gao and Waechter (50) have also pointed out in their research that users' trust in mobile payments is directly influenced by their trust in online payments, which is actually the process of trust transfer between the network and mobile environments. In the medical field, this phenomenon of trust transfer is also worthy of attention. With the rise of Internet medicine, more and more patients begin to communicate and consult with doctors through online platforms. This online interaction provides patients with a convenient and efficient medical service experience, while also establishing a preliminary trust relationship between them and doctors. However, when patients require further treatment or examination, they may still need to go to the hospital for face-to-face communication with doctors. In this situation, whether the trust of patients in doctors online can be smoothly transferred to offline has become a worthwhile research question.

We know that in the field of Internet medicine, online trust is usually established based on a number of factors, including platform reputation (platform popularity, reputation and user evaluation, etc.), doctor qualifications (doctor's education, professional title, professional experience, etc.), interactive experience (online communication quality and response between patients and doctors, etc.), and information security (patient's personal information protection and data security, etc.), which together constitute the basis for patients to trust doctors or platforms in the online environment.

When patients require further treatment or examination, they often hope to transfer the trust established online to the offline environment. The demand for this transfer stems from patients' expectations for offline medical services, including confirmation of professional abilities, development of treatment plans, and establishment of emotional connections. However, the process of transferring trust from online to offline is not simply a continuation, but is based on a series of complex causal mechanisms. These mechanisms include: consistency of information, consistency of service experience, satisfaction of patient expectations, social identity and sense of belonging, etc. Specifically, patients will pay attention to whether the information provided by doctors in online and offline environments is consistent, including disease diagnosis, treatment plans, etc., whether the quality, attitude, professionalism, etc. of online and offline services are consistent, whether offline services can meet patients' expectations and needs, such as treatment effectiveness, communication quality, etc., and whether patients can gain a sense of social identity and belonging through communication with doctors in offline environments.

Based on the above analysis, we can see that the establishment of online trust provides a preliminary trust foundation for patients. This foundation is established based on factors such as doctors' professional competence, communication skills, and the convenience and safety provided by the platform. When there is a correlation between online and offline environments, patients are more likely to transfer online trust to the offline environment. This correlation is mainly reflected in the consistency of doctor identity, continuity of information, and coherence of service experience. The quality and experience of offline services further consolidate the trust established online. When consistency is maintained between online and offline environments, patients are more likely to transfer online trust to the offline environment.

Therefore, this article makes the following assumptions:

H1: Patients' offline trust will be positively influenced by their online trust.

### 3.2 Reputation feedback

On e-commerce platforms, reputation mechanisms are crucial for the survival and development of merchants. Under the influence of online reputation, consumers and merchants from different geographical locations can quickly connect. Posting feedback information such as digital ratings and text comments is the most traditional reputation mechanism. After the system summarizes and organizes the feedback information, it will be included in the reputation files of merchants and consumers, providing a basis for user decision-making. Similarly, the influencing factors of offline trust also include reputation mechanisms. Racherla and Friske (51) conducted in-depth analysis on the role of reputation from both online and offline markets, revealing the commonalities and differences of reputation in different environments. The reputation mechanism can also affect users' trust in online medical platforms. In order to attract users and encourage more users to choose the platform, medical service platforms will regulate their own medical behavior, continuously improve the level and quality of medical services, and gain a good online reputation. As an intangible asset of traders, reputation can effectively constrain their behavior, greatly reducing the likelihood of moral hazard and adverse selection. Through research, scholars represented by Noort have found that managing reputation is not easy, but once lost, the impact can be profound. Consumers are more susceptible to the impact of negative transactions (52). Many studies have confirmed that the higher the credibility of a business, the higher the trust of consumers in it. In any field and market, reputation is one of the key factors in winning trust (53). For doctors, as a special service provider, their online reputation can also affect patients' trust in them. This impact is not only reflected in online consultation and interaction, but also extends to the offline medical process. Therefore, doctors need to value their online reputation and win the trust and respect of patients by providing high-quality services and positive interactions. Therefore, this article makes the following assumptions:

H2: Patients' online trust will be positively influenced by doctors' online reputation.
H3: Patients' offline trust will be positively influenced by doctors' online reputation.

The transfer of trust between multiple contexts is not a simple psychological transfer, but a complex process. Only with specific characteristics or structures can trust be transferred in different contexts. If there are common factors that provide guarantees for different situations, the transfer of trust will become more natural and smooth (54). For example, if the trust relationship in one context is based on professionalism, reliability, and transparency, and these factors also exist in another context, then individuals are more likely to transfer trust from one context to another. This phenomenon of trust transfer provides us with a window into the essence of trust (55). In the process of trust transfer, reputation plays a crucial role as an observable signal. Reputation is not only an evaluation of a target's past behavior, but also a signal that indicates that the target possesses certain advantages or traits (56). In the medical field, if a doctor has established a good reputation on online platforms, patients are more likely to extend this trust to offline environments (57). On the one hand, reputation can reflect a doctor's level of service ability, providing patients with objective information about the doctor's professional competence and service quality. On the other hand, reputation can also promote the reduction of uncertainty in patients' use of the platform. When patients are faced with numerous choices, they often tend to choose doctors with higher reputations because these doctors can provide them with more reliable and professional services. Based on the above discussion, we make the following assumptions:

H4: The relationship between patient trust online and offline will be positively moderated by the online reputation of doctors.

### 3.3 Research model

**Figure 1:**
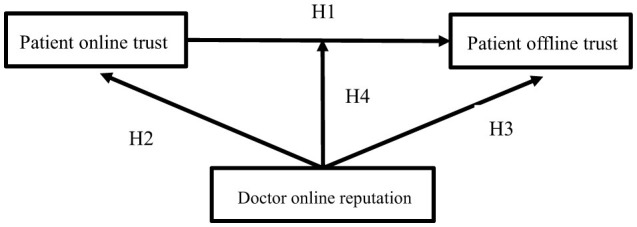
Research model.

We have constructed an online and offline trust model for patients based on the assumptions mentioned above, as shown in Figure 1.

## 4 Method

### 4.1 Variables

#### 4.1.1 Dependent variable and independent variable

(1) Patient offline trust

This variable is the main dependent variable in this study, as it reflects the patient's decision to ultimately choose a doctor for offline treatment. Trust, as a complex social psychological phenomenon, is often difficult to quantify directly, but we can infer patients' level of trust from their behavior (58). In this study, we assume that the behavior of patients choosing specific doctors for offline consultations is a direct reflection of their trust in doctors. To quantify patients' offline trust, we chose the total number of offline appointments received by doctors as the measurement indicator. This is because only when patients have a high level of trust in the doctor's professional ability, service attitude, patient evaluation, and other aspects, will they choose this doctor for offline treatment when needed. The number of offline appointments not only represents the actual behavioral choices of patients, but also indirectly reflects their level of trust in the doctor.

The offline appointment volume of patients on online medical platforms refers to the behavior of patients agreeing with doctors through online platforms to receive face-to-face diagnosis and treatment services at specific times and locations during a specific time period. This behavior usually occurs when the patient has a preliminary understanding or experience of the doctor's services and decides to further receive their professional treatment or health guidance. In the Good Doctor Medical Platform, the total number of consultations on this front line is automatically calculated and displayed to users on the platform's webpage. This indicator can be directly obtained through the online medical platform webpage.

(2) Patient online trust

Patient online trust plays an important role in this study, serving as both the dependent variable in the early stages and a significant factor influencing patient offline trust. On online medical platforms like “Good Doctor,” patients can gain a preliminary understanding of doctors by browsing their personal information, historical evaluations, professional expertise, and other information, and establish trust in doctors based on this information (58). When a patient chooses to initiate an online consultation with a doctor, this behavior itself can be seen as a manifestation of the patient's trust in the doctor online. The more online consultations there are, the more patients choose this doctor for consultation, indirectly reflecting the popularity of doctors on online platforms and the level of trust among patients. In order to quantify patients' online trust, we chose the total number of doctors' online consultations as the measurement indicator.

The online consultation volume of patients on an online medical platform refers to the total number of patient consultations received by a doctor on the platform during a specific time period. The “online consultation” referred to here includes all online medical behaviors such as disease consultation, health guidance, and online consultation. This quantity reflects the level of patient acceptance of a certain doctor's service in online healthcare. In the Good Doctor Medical Platform, the total number of consultations on this front line is automatically calculated and displayed to users on the platform's webpage. This indicator can be directly obtained through the online medical platform webpage.

(3) Doctor's online reputation

Patient online comments reflect the true experience and satisfaction of patients, which is an important indicator for measuring the quality and reputation of doctors' services (59). Patients share their medical experiences through online platforms such as medical forums, health communities, or specialized doctor evaluation websites, and evaluate the doctors' service attitude, professional level, treatment effectiveness, and other aspects. These comments are not only a direct reflection of patients' real experiences and satisfaction, but also provide valuable reference information for other patients, thereby affecting their trust and choice of doctors (60). The importance of doctors' online reputation is self-evident. If a doctor has numerous positive and detailed online reviews, it often means that he has gained recognition and trust from a large number of patients (61). The wide coverage and real-time nature of online comments are its unique advantages in measuring doctors' online reputation. With the popularity of the Internet, the real-time nature of online comments enables doctors' reputation to quickly update and reflect the latest patient evaluations, providing timely and accurate data for research. Therefore, this study measured the online reputation of doctors using the content of patients' online comments.

#### 4.1.2 Control variables

In order to more accurately study the relationship between patient trust and doctors' online reputation, we need to effectively identify and control other factors that may affect patient trust (62). In this study, there are two variables: hospital level and doctor title. The title of doctor is an important criterion for measuring the professional level and experience of doctors. In this study, we mainly focused on the clinical titles of doctors and divided them into four levels: chief physician, associate chief physician, attending physician, and resident physician. Meanwhile, as the vast majority of doctors are at the level of chief physician and associate chief physician, and the proportion of physicians at other levels in the sample is extremely low, we focus on chief and associate chief physicians (Title1 = associate chief physician, Title2 = chief physician, measured using 0–1 variables). Hospital level is another important control variable. In China, hospital levels are usually divided into Grade 3, Grade 2, and Grade 1 (Grade 1 = Grade 1 hospital, Grade 2 = Grade 2 hospital, Grade 3 = Grade 3 hospital, with corresponding measurement values of 0, 1, and 2). Table 1 shows the meanings and measurement methods of different variables.

**Table 1:** Variable description and measurement.

**Variable**	**Describe**	**Symbol**	**Measure**
Patient offline trust	Patient offline trust behavior	OFT	Number of patients who make offline appointments with doctors
Patient online trust	Patient online trust behavior	ONT	Number of patients who consult doctors online
Professional competence	Extract content from comment texts	PC	Mentioning this feature: 1, otherwise 0 Extract the sum of features
Service attitude	Extract content from comment texts	TE	Mentioning this feature: 1, otherwise 0 Extract the sum of features
Treatment effect	Extract content from comment texts	SA	Mentioning this feature: 1, otherwise 0 Extract the sum of features
Doctor's title	Doctor's clinical title	Title	Title 1 and Title 2 represent the deputy director and chief physician, respectively, with a measurement variable range of [0, 1]
Hospital level	The level of the hospital where the doctor is located	Grade	Grade 1 = First level hospital Grade 2 = Second level hospital Grade 3 = Third level hospital, Variable range of [0–2]

Among the seven variables mentioned above, the four variables of patient offline trust, patient online trust, doctor title, and hospital level can be relatively easily obtained from online medical platforms through direct data statistics. Specifically, patient offline trust can be quantified by the number of offline appointments obtained by doctors; Patient online trust can be measured by counting the number of online consultations received by doctors; the doctor's professional title and hospital level can be directly obtained from the doctor's personal page information. However, the three variables of professional competence, service attitude, and treatment effectiveness are not so easily obtained directly. In this study, we extracted and measured online comment texts through analysis and mining. This is because patient reviews often include multiple aspects of their evaluation of the doctor, including the accuracy of the doctor's diagnosis, the rationality of the treatment plan, surgical skills, communication methods, service attitude, etc.

In the following research, we will provide a detailed introduction to the feature mining and mapping methods of online user comments. The main purpose of this method is to extract features related to doctors' professional abilities, service attitudes, and treatment outcomes from a massive amount of patient comments. Specifically, we will use natural language processing (NLP) and text mining techniques to perform segmentation, part of speech tagging, sentiment analysis, and other processing on comment texts, in order to extract keywords, phrases, or sentences related to professional competence, service attitude, and treatment effectiveness (63). Then, we quantify these extracted features, such as calculating the frequency of keywords or phrases, emotional tendencies, etc., to evaluate the performance of doctors in these areas. In this way, we can transform the three variables of professional competence, service attitude, and treatment effectiveness that were originally hidden in the comment text into observable and quantifiable indicators, thereby more comprehensively evaluating the performance of doctors and the trust level of patients.

### 4.2 Feature mining and mapping methods

#### 4.2.1 Feature mining

The latent Dirichlet distribution reveals the similarity between elements in observed data sets (such as document sets) by introducing hidden layers (i.e., latent themes or topics) (64). As an unsupervised machine learning method, it has been widely applied in natural language processing. The core idea of LDA is to assume that each document is a mixture of multiple potential topics, and each topic is composed of a series of words in the vocabulary according to a certain probability distribution. When faced with a large amount of online user comment data, LDA can help us effectively mine the implicit topic features in the comments. These thematic features often reflect users' overall evaluation and focus on a certain product or service. In order to better utilize LDA for feature mining of comments, we need to preprocess the original comment text. The first step in preprocessing is to convert the comment text into a Bag of Words (BOW) pattern (65). When converting comment text into bag of words mode, we first need to segment the text. Due to the lack of clear separators between Chinese words, word segmentation is an essential step in Chinese text processing. Through word segmentation, we can divide continuous Chinese text into discrete word units, laying the foundation for subsequent text representation and analysis. After the word segmentation is completed, we need to further clean the text data. The purpose of this step is to remove noisy information from the text, such as stop words (common but meaningless words like “de” and “is”), special characters, HTML tags, etc. Through data cleaning, we can improve the quality of text and enable LDA models to more accurately capture thematic information in the text. After processing the comment text collection through a word segmentation tool, a valuable set of text words *w*_*ij*_ is formed.


(1) $$\begin{array}{l} x_{i}=\left\{w_{i1},.....w_{ij},....\right\} \end{array}$$

**Figure 2:**
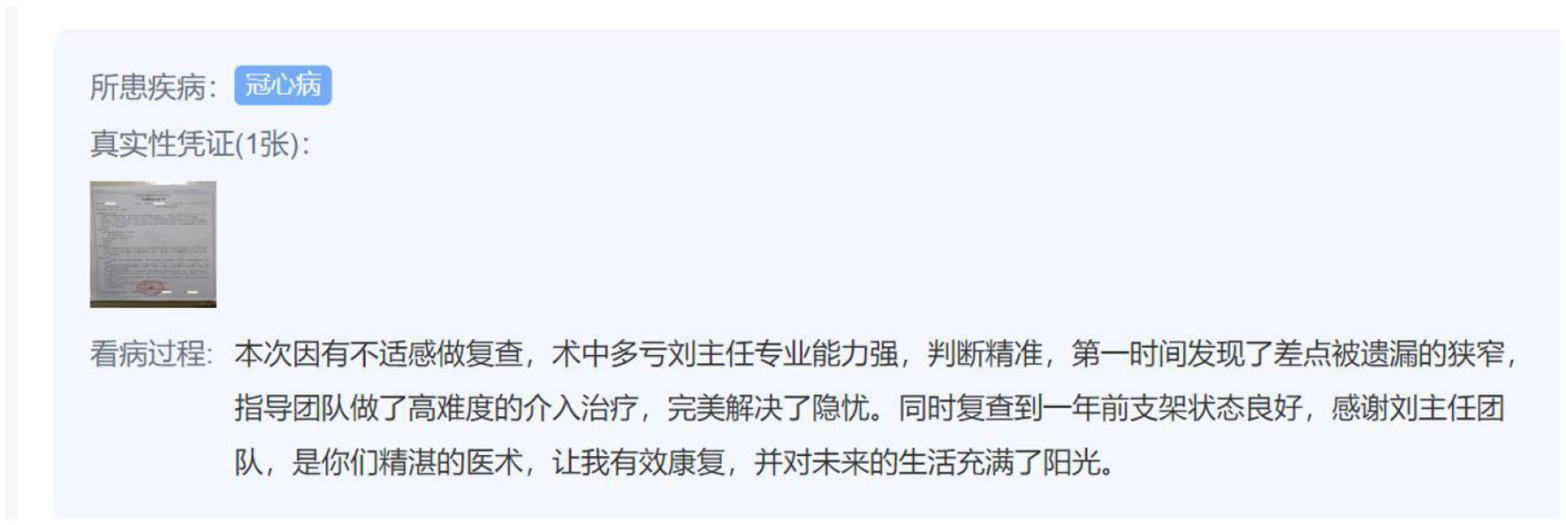
Example of online user comment retrieval page.

For example, as shown in Figure 2, given an online review of a coronary heart disease patient, if the content of the review posted by the reviewer *x*_1_= “Due to discomfort, I underwent a follow-up examination. Thanks to Director Liu's strong professional ability and accurate judgment, I was able to detect the narrow area that was almost missed in the first place. I guided the team to perform a difficult intervention treatment, which perfectly solved the hidden problem. At the same time, it was rechecked and found that the stent was in good condition a year ago. Thank you to Director Liu's team for your superb medical skills, which enabled me to effectively recover and fill my future life with sunshine.” We can intuitively see that multiple characteristic words are mentioned in this comment, so we can conclude that *x*_1_= {Strong professional ability, accurate judgment, timely detection, high difficulty intervention treatment, perfect solution to hidden worries, good condition, superb medical skills, effective rehabilitation}.

Then, we analyze the set of comment data *X* = {*x*_*i*_} Using latent topic model LDA algorithm for feature content mining, generating a series of feature sets *T* = {*T*_*j*_}:


(2) $$\begin{array}{l} T_{j}=\left\{t_{j1},.....t_{jk},....\right\} \end{array}$$

Among them, *t*_*jk*_ is the feature word associated with the feature *T*_*j*_. Table 2 presents the extracted features and examples of feature words.

**Table 2:** Features and related vocabulary.

***T*_j_**	**Feature**	**Related feature words**
*T*_1_	Professional competence	For example: strong professional ability, accurate judgment, superb medical skills, high difficulty intervention therapy
*T*_2_	Service attitude	For example, discovering at the first time
*T*_3_	Treatment effect	For example: perfectly resolved hidden concerns, in good condition, and effectively recovered

#### 4.2.2 Mapping method

When dealing with massive online user comments, directly applying data mining algorithms for feature mining often faces the challenge of data sparsity. Data sparsity is a common issue, especially in the field of text processing, which is mainly caused by the following factors: firstly, due to the diversity of commenters' backgrounds, their educational level, motivation for posting comments, and personal habits can all affect their word choice. This leads to a diversity of word expressions in the comment text, which in turn makes the distribution of features sparse (66). For example, for the same service or product, different reviewers may use completely different vocabulary to describe their experiences and feelings. Secondly, there are a large number of synonyms or synonyms in language, which, although expressing similar meanings, are regarded as different features in the text. This further exacerbates the sparsity of feature data. This situation is particularly evident in online medical reviews, as reviewers may use different vocabulary to describe the same medical experience or emotion. Furthermore, online user comments, as a form of emotional expression, typically have a more free and colloquial writing style. Although this expression is intuitive and easy to understand, it also leads to a large amount of non-standard vocabulary and expressions in the text. At the same time, due to the medical field involved in the comments, it is inevitable to involve some professional terms, such as disease types, surgical names, and medication situations. The emergence of these professional terms further increases the complexity of feature data.

In order to address the lack of feature reflecting data in massive comments, we adopted the method of mapping features through comments. Its core lies in achieving the transformation from text to feature set based on the semantic distribution of words. Regarding the dataset ∪{*x*_*i*_}, If the conditions are met |*T*_*j*_∩*x*_*j*_|≥0, it is believed that features were mentioned in the comment text *x*_*i*_.

**Figure 3:**
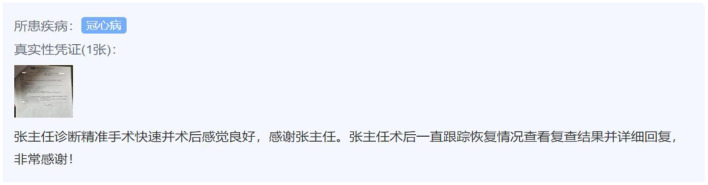
Example of online user comment retrieval page.

For example, as shown in Figure 3, given a comment text *x*_2_= “Dr. Zhang's diagnosis was accurate, the surgery was performed quickly, and the postoperative feeling was good. Thank you to Dr. Zhang. Dr. Zhang has been tracking the recovery situation after the surgery, reviewing the results of the follow-up examination, and providing detailed responses. Thank you very much,” By using the “comment feature” mapping method, then *x*_1_ can be expressed as: *x*_1_= {Accurate diagnosis, fast surgery, good postoperative feeling, continuous tracking of recovery after surgery, detailed response}. Based on the above method, we obtain a feature set that includes service attitude, treatment effectiveness, and professional competence. Based on the feature set, we can convert text statements into comment feature vectors.

### 4.3 Data collection

The data sample of this article comes from “Haodafu,” the largest online medical platform in China. The platform not only provides online services such as graphic and textual consultations, but also offers offline appointment functions, which allows us to simultaneously examine the trust status of doctors both online and offline. We can calculate online trust by counting the number of patients received by doctors online, and predict offline trust based on the number of appointments with doctors offline. This dual dimensional trust assessment method can more comprehensively and accurately reflect the trust relationship between doctors and patients.

In order to ensure the accuracy and completeness of the data, we have adopted a web data scraping program (crawler) to automatically collect relevant information from the online website of Good Doctor. By programming specific rules and parameters, crawlers can simulate user browsing behavior, automatically capture and organize the data we need, such as doctor information and patient evaluations. It is worth noting that although the platform provides both online and offline medical services, the number of offline consultations is much lower compared to online consultations. These doctors usually conduct related medical activities online while conducting offline diagnosis and treatment. We should focus on these types of doctors as the main research subjects. This choice helps us to have a more comprehensive understanding of the trust status of doctors in different service scenarios, as well as the correlation and differences between these trust statuses.

**Figure 4:**
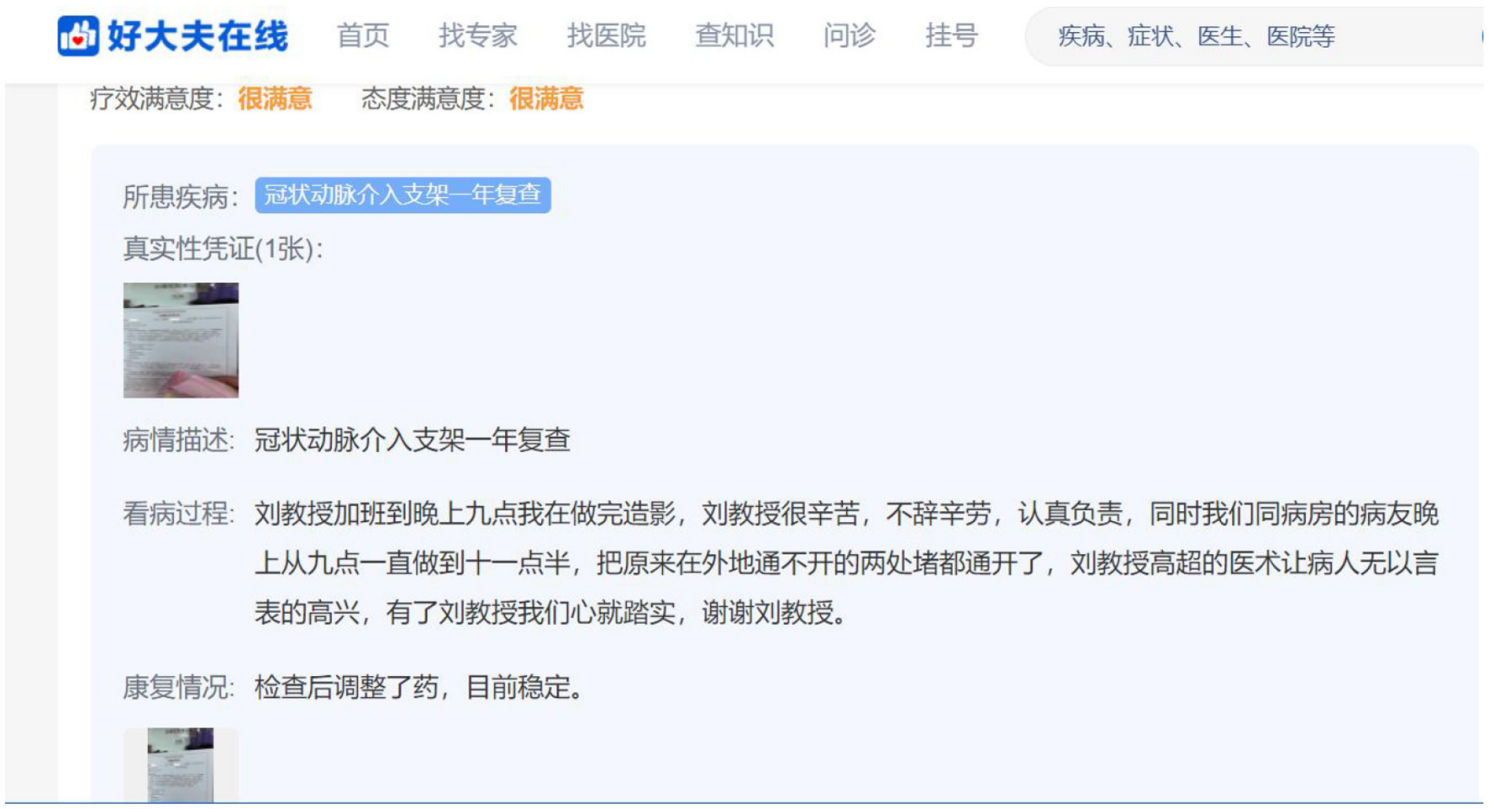
Example of capturing user comments from good doctor online.

As shown in Figure 4, the above picture is the online comment interface of Good Doctor that we randomly captured. Here, *X* represents the collection of captured comments, and *x*_*i*_ϵ*X*, (*i* = 1, ...., |*X*|) represents the comment of *i*.

When conducting this study, we initiated the collection of doctor data on July 1, 2022. Based on the specific needs of the research, we first collected the website links of all doctors who provided online appointment services on that day. Subsequently, we used these URLs to automatically access the doctor's homepage and extracted their homepage information. Next, we conducted a detailed screening based on the completeness of the homepage information and whether it contained user comment text, to ensure that the final obtained doctor data was comprehensive and met our research standards.

### 4.4 Distribution

When processing and analyzing raw comment texts captured from the “Good Doctor” online medical platform, we face a difficult challenge, which is that these comment data do not have clear labels or clues to directly identify the key features of user discussions. However, the rich information contained in these comments is crucial for understanding patients' needs, doctors' service quality, and the trust relationship between doctors and patients. In order to extract valuable information from a massive amount of comment texts, we used a Java version based on LDA (Latent Dirichlet Allocation) to extract features from 597,623 comments. LDA is an unsupervised machine learning algorithm used to discover hidden themes or features from a collection of texts (67). However, a major limitation of LDA is the need to pre-set two key parameters before running: “number of features” and “number of associated words for each feature.”

In traditional LDA method applications, the setting of these two parameters usually depends on the researcher's experience or experimental attempts. In this study, a series of experiments were conducted to ensure that the mined features fully reflect the essence of the comment data. We set multiple feature numbers ranging from 1 to 20 and used the perplexity metric to evaluate the performance of the LDA model under different parameters. Confusion is an indicator that measures the degree to which a model fits text data, with a smaller value indicating better model performance. After multiple repeated experiments, we found that when the number of features is set to 3, the perplexity of the LDA model reaches its lowest level, which means that the model can best capture hidden features in the comment data. Therefore, we chose 3 as the most suitable number of features.

However, relying solely on unsupervised LDA algorithms to mine features in comments may have limitations. In order to further improve the accuracy and completeness of feature mining, we introduced a semi supervised method (68). Specifically, we used a word vector based representation method that can capture the semantic relationships between words. By calculating the semantic similarity between the feature related words mined by LDA and other words, we can select words that are similar to these feature words, thereby expanding the range of feature related words. In order to ensure that each feature is sufficiently complete, we utilized manual adjustment to further improve the feature related words, and achieved the transformation from text to feature word set through mapping. If there are words with associated features in the comments, set the value corresponding to that feature to 1; otherwise, set it to 0. Through this approach, we can quantitatively evaluate the level of mention of each feature in each comment. Finally, we accumulated and summarized the feature word frequencies of all doctors, and obtained the total numerical values of each doctor on each feature. This step not only helps us understand the performance of doctors in different aspects, but also provides an important data foundation for subsequent analysis and evaluation. Table 3 presents the characteristics of online user comment content and typical characteristic words used to express this feature.

**Table 3:** Features mentioned in online comment texts.

**Feature**	**Typical characteristic words (top 5)**
Professional competence	Highly skilled, accurate in judgment, exquisite, professional, efficient
Service attitude	Responsible, meticulous, patient, caring, respectful
Treatment effect	Recovery, rehabilitation, restoration of health, stability, improvement of condition

## 5 Data

### 5.1 Statistical analysis

We also collected and analyzed data related to the personal pages of doctors on the Good Doctor online platform. Table 4 shows the specific analysis results.

**Table 4:** Descriptive statistical analysis of variables.

**Variable**	**Minimum value**	**Maximum value**	**Mean value**	**Standard deviation**	**Median**
OFT	0	17,065	426.54	587.25	206
ONT	2	49,342	2,768.86	3,986.12	1,428
PC	0	6,232	42.56	142.48	27
SA	0	8,965	71.24	205.32	19
TE	0	10,324	608.45	827.65	249

When conducting statistical analysis on the dataset, we noticed that the data distribution presented in Table 4 exhibits skewness. In order to ensure the accuracy and effectiveness of data analysis, we have decided to implement logarithmic processing on all variables involved to correct this skewed distribution. Logarithmic processing is a commonly used data conversion method that can effectively reduce data skewness, making the originally skewed data closer to a normal distribution, thereby making subsequent data analysis more accurate and reliable. To evaluate whether the logarithmically transformed variables are affected by multicollinearity, we calculated the variance inflation factor (VIF) for each variable. VIF is an important indicator for measuring the severity of multicollinearity, and it is generally believed that multicollinearity problems are not significant when the VIF value is < 10. According to our calculations, the VIF values of all variables are below 10, indicating that the issue of multicollinearity between variables is not severe after logarithmic transformation and will not have a significant impact on our research (69).

### 5.2 Model

Patient online and offline trust are the focus of this study, as they are continuous dependent variables. Based on this, we chose multiple linear regression method for data analysis. In order to explore in depth how the independent variable and a potential moderating effect affect these two dependent variables, we constructed three models with the following specific formulas:


(3) $$\begin{array}{c} \;\ln\;ONT=\alpha_{0}+\alpha_{1}Grade1+\alpha_{2}Grade2+\alpha_{3}Grade3\\ +\alpha_{4}Title1+\alpha_{5}Title2+\alpha_{6}\ln\;PC+\alpha_{7}\ln\;SA+\alpha_{8}\ln\;TE+\varepsilon \end{array}$$

(4) $$\begin{array}{c} \;\ln\;OFT=\beta_{0}+\beta_{1}Grade1+\beta_{2}Grade2+\beta_{3}Grade3\\ +\beta_{4}Title1+\beta_{5}Title2+\beta_{6}\ln\;PC+\beta_{7}\ln\;SA+\beta_{8}\ln\;TE\\ \;+\beta_{9}\ln\;ONT+\mu \end{array}$$

(5) $$\begin{array}{c} \ln\;OFT=\gamma_{0}+\gamma_{1}Grade1+\gamma_{2}Grade2+\gamma_{3}Grade3\\ \;+\gamma_{4}Title1+\gamma_{5}Title2+\gamma_{6}\ln\;PC+\gamma_{7}\ln\;SA\\ \;+\gamma_{8}\ln\;TE+\gamma_{9}\ln\;ONT+\gamma_{10}\ln\;PC\times \ln\;ONT\\ +\gamma_{11}\ln\;SA\times \ln\;ONT+\gamma_{12}\ln\;TE\times \ln\;ONT+\theta \end{array}$$

Model 1: In this model, we focus on the dependent variable—patient online trust. To more accurately predict patient online trust, we included several key variables. Firstly, professional competence, service attitude, and treatment effectiveness are set as explanatory variables, which directly reflect the performance of doctors in medical services. In addition, we also considered hospital level and doctor title as control variables, which may affect patients' trust in doctors' online services.

Model 2: Similar to Model 1, Model 2 also explores the relationship between doctor performance and patient trust. However, in this model, we shifted our focus to patient offline trust. In addition to the three explanatory variables of professional competence, service attitude, and treatment effectiveness, we also specifically included patients' online trust as an explanatory variable to examine the impact of online trust on offline trust. Hospital level and doctor's professional title are still included as control variables.

Model 3: This model is a further extension of Model 2. In addition to the original explanatory and control variables, we have also added corresponding interaction terms, which are composed of the multiplication of professional competence, service attitude, treatment effectiveness, and online trust variables. The design of interaction items aims to explore the possible interactions between these factors and how they collectively affect the level of patient offline trust.

### 5.3 Result analysis

Table 5 shows the specific results of regression analysis. Patient trust model 1 only has control variables, while model 2 has added explanatory variables. After adjustment, the latter *R*^2^ = 0.689, that is to say, patients' online trust can be explained 68.9% by controlling for variables and independent variables. In patient offline model 1, there are only control variables. In model 2, explanatory variables have also been added. In model 3, in addition to control and explanatory variables, we have also added interaction terms. The results showed that the adjusted values *R*^2^ for models 2 and 3 were 0.597 and 0.648. That is to say, patient offline trust can be explained 59.7% by controlling for variables and independent variables, and adding interaction terms on this basis, patient offline trust can be explained 64.8%. The above models all have good fit and interpretability.

**Table 5:** Results of multiple linear regression.

**Variable**	**Patient online trust**		**Patient offline trust**		
	**Model 1**	**Model 2**	**Model 1**	**Model 2**	**Model 3**
Constant	5.379^***^ (0.078)	3.295^***^ (0.054)	4.512^***^ (0.091)	−1.093^***^ (0.157)	−0.64^***^ (0.283)
Third level hospital (Grade 1)	0.242^***^ (0.062)	0.151^***^ (0.024)	0.341^**^ (0.059)	0.339^**^ (0.046)	0.237^***^ (0.054)
Second level hospital (Grade 2)	0.124^**^ (0.059)	0.158^**^ (0.024)	0.119^***^ (0.048)	0.258^**^ (0.042)	0.160^**^ (0.051)
First level hospital (Grade 3)	−0.063^**^ (0.067)	−0.144^**^ (0.035)	−0.285^*^ (0.076)	−0.200 (0.063)	−0.197^**^ (0.058)
Chief Physician (Title 1)	0.164^**^ (0.085)	0.158^***^ (0.034)	0.149^***^ (0.087)	0.413^***^ (0.064)	0.436^***^ (0.064)
Deputy Chief Physician (Title 2)	−0.045 (0.078)	−0.148 (0.038)	0.291^*^ (0.093)	0.286^*^ (0.061)	0.276^***^ (0.061)
Patient online trust (ONT)				0.634^***^ (0.027)	0.564^***^ (0.043)
Professional competence (PC)		0.053^***^ (0.008)		0.032 (0.064)	0.051 (0.048)
Service attitude (SA)		0.198^***^ (0.008)		0.102^**^ (0.015)	0.115^**^ (0.015)
Treatment effect (TE)		0.596^***^ (0.008)		0.131^***^ (0.012)	0.218^***^ (0.012)
PC × ONT					0.009 (0.038)
SA × ONT					0.012^**^ (0.008)
TE × ONT					0.029^**^ (0.008)
*N*	4,526	4,526	4,526	4,526	4,526
Adjusted *R*^2^	0.012	0.689	0.034	0.597	0.648
*F*	3.56^***^	707.13^***^	37.40^***^	423.60^***^	381.87^***^

^*^p < 0.05.

^**^p < 0.01.

^***^p < 0.001.

Observing the Table 5, it can be seen that Model 2 of online trust has a significant regression coefficient, and professional competence, service attitude, and treatment effectiveness all have a significant impact on patients' online trust. It is worth noting that the impact of treatment effect is the greatest (β = 0.596, *p* < 0.001), which strongly indicates that treatment effect is the most important factor for patients to evaluate doctors online. In contrast, the influence of professional competence is the smallest (β = 0.053, *p* < 0.001), indicating that professional competence also has a significant impact on patients' online trust, but its degree of influence is relatively small. It is interesting that the influence of service attitude (β = 0.198, *p* < 0.001) even exceeds professional competence. This finding reveals that in the modern medical environment, patients have very high expectations and requirements for doctors' service attitude, which greatly affects patients' online trust in doctors.

According to the explanatory power of offline trust model 2, it can be found that patients' offline trust is directly affected by the treatment effect of doctors and online trust, especially the influence value of offline trust has reached 0.634, indicating a particularly significant impact. This means that patients often form initial trust in doctors through online channels before engaging in offline communication, which will directly affect the effectiveness of offline communication and patient satisfaction. In addition, besides the insignificant effect of professional competence on patients' offline trust, service attitude has a significant impact on patients' offline trust (β = 0.102, *p* < 0.01), and treatment effectiveness also has a significant impact on patients' offline trust (β = 0.131, *p* < 0.001). This indicates that the service attitude of doctors is an important factor for patients to consider when evaluating offline trust. A good service attitude can increase patients' comfort and trust, thereby improving their satisfaction and trust in doctors' offline services. Meanwhile, the treatment effect remains one of the most concerning aspects for patients, as it directly affects their health and recovery. When the doctor's treatment effect is significant and can effectively solve the patient's health problems, patients will naturally have higher trust and satisfaction with the doctor. Therefore, the treatment effect is also an important factor that patients cannot ignore when evaluating offline trust.

In Model 3 of patient offline trust, we specifically focused on the impact of interaction terms to reveal possible interactions between different factors. Firstly, it is worth noting that in addition to professional competence, service attitude (β = 0.012, *p* < 0.01) and treatment efficacy (β = 0.029, *p* < 0.01) both showed significant positive effects in the interaction terms of Model 3. This indicates that when patients perceive doctors to have a positive and friendly service attitude, they are more likely to trust doctors in the offline environment. The enhancement of this sense of trust not only comes from patients' online trust, but is also deeply influenced by doctors' service attitude. Similarly, the therapeutic effect plays a crucial role in the formation of offline trust among patients. Patients often use the treatment effect as a key indicator to evaluate the ability of doctors and the quality of medical services. When the treatment effect is significant, patients are more likely to trust doctors and are willing to continue receiving their offline medical services. In addition, the explanatory power of Model 3 has improved compared to previous models, mainly due to the introduction of interaction terms. The introduction of interaction terms enables us to comprehensively consider the interactions between different factors, thereby more accurately predicting changes in patient offline trust. This discovery not only validates some hypotheses about the moderating effect of doctors' online reputation, but also provides us with a comprehensive understanding of the mechanism of patient trust formation. By comparing the regression results of different models, we can see more clearly the impact and mechanism of each factor on patient trust, providing useful references for future medical services and patient trust management. Table 6 details the hypothesis test results proposed in this paper.

**Table 6:** Regression coefficients and hypothesis testing results.

**Hypothetical relationship**	**Results**
Patient online trust → patient offline trust	Accept
Professional competence → patient online trust	Accept
Service attitude → patient online trust	Accept
Treatment effect → patient online trust	Accept
Professional competence → patient offline trust	Reject
Service attitude → patient offline trust	Accept
Treatment effect → patient offline trust	Accept
Professional ability × patient online trust → patient offline trust	Reject
Service attitude × patient online trust → patient offline trust	Accept
Treatment effect × patient online trust → patient offline trust	Accept

^*^p < 0.05.

^**^p < 0.01.

^***^p < 0.001.

### 5.4 Robustness test

In the initial data collection, we only obtained cross-sectional data at the same time point. Although these data revealed key factors affecting patient trust, the characteristics of cross-sectional data made it difficult for us to accurately evaluate the dynamic effects of these influencing factors over time. In order to gain a more comprehensive understanding of the long-term impact of these factors on patient trust, we collected sample data again on August 1, 2022, 1 month after the initial data collection. The doctors who provide offline diagnosis and treatment appointment services may change at different times. In order to ensure that the two rounds of data can match each other, we have processed the data appropriately. After screening, we successfully retained the data of 4,235 doctors corresponding to the first round of data. In order to more accurately evaluate the changes in the quality of doctor services and patient trust in a short period of time, we calculated the increment of online consultations and offline appointments during the month as a unit time. This indicator can intuitively reflect the improvement or decline of doctor service quality, as well as the trend of changes in patient trust. After incorporating these two incremental dependent variables into the model for the second round of analysis, we obtained the results shown in Table 7.

**Table 7:** Results of robustness test.

**Variable**	**Patient online trust**		**Patient offline trust**		
	**Model 1**	**Model 2**	**Model 1**	**Model 2**	**Model 3**
Constant	5.418^***^ (0.078)	3.385^***^ (0.057)	4.632^***^ (0.091)	−1.087^***^ (0.164)	−0.83^***^ (0.292)
Third level hospital (Grade 1)	0.265^***^ (0.067)	0.184^***^ (0.032)	0.335^**^ (0.054)	0.341^**^ (0.052)	0.242^***^ (0.062)
Second level hospital (Grade 2)	0.136^**^ (0.061)	0.162^**^ (0.026)	0.121^***^ (0.052)	0.262^**^ (0.044)	0.154^**^ (0.063)
First level hospital (Grade 3)	−0.059^**^ (0.063)	−0.156^**^ (0.025)	−0.292^*^ (0.074)	−0.208 (0.059)	−0.199^**^ (0.064)
Chief Physician (Title 1)	0.174^**^ (0.079)	0.161^***^ (0.032)	0.152^***^ (0.085)	0.425^***^ (0.068)	0.454^***^ (0.078)
Deputy Chief Physician (Title 2)	−0.049 (0.081)	−0.157 (0.036)	0.302^*^ (0.094)	0.292^*^ (0.065)	0.252^***^ (0.056)
Patient online trust (ONT)				0.641^***^ (0.024)	0.572^***^ (0.038)
Professional competence (PC)		0.053^***^ (0.009)		0.045 (0.029)	0.009^*^ (0.015)
Service attitude (SA)		0.201^***^ (0.009)		0.112^**^ (0.012)	0.134^**^ (0.012)
Treatment effect (TE)		0.572^***^ (0.009)		0.129^***^ (0.012)	0.225^***^ (0.012)
PC × ONT					0.012 (0.032)
SA × ONT					0.018^**^ (0.009)
TE × ONT					0.030^**^ (0.009)
*N*	4,235	4,235	4,235	4,235	4,235
Adjusted *R*^2^	0.397	0.678	0.402	0.502	0.552
*F*	3.62^***^	710.23^***^	37.40^***^	433.51^***^	383.84^***^

^*^p < 0.05.

^**^p < 0.01.

^***^p < 0.001.

According to the analysis results, it can be found that the of model is at a high level, with strong explanatory power, and can effectively predict changes in patient trust and doctor service performance. We found that in the second round of analysis, the impact of professional competence on patients' offline trust changed from insignificant to significant (β = 0.009, *p* < 0.05). This change may indicate that as time goes on and interaction between patients and doctors deepens, patients begin to pay more attention to the professional abilities of doctors. The improvement of professional skills can enhance patients' trust in doctors, thereby promoting an increase in the number of offline appointment registrations. The impact of other independent variables on patient trust and doctor service performance, apart from professional competence, remains consistent with the results of the first round of analysis. This result validates the robustness of our previously proposed hypothesis and model, demonstrating that the impact of these factors on patient trust is consistent across different time periods. Therefore, the robustness of the results of this study has been validated.

## 6 Discussion

### 6.1 Research conclusion

In the context of rapid development of digital healthcare, the interaction mode between patients and doctors is undergoing profound changes, and online medical consultation and services have become an important channel for patients to obtain medical information and treatment advice. However, the uncertainty brought by the online environment and the problem of information asymmetry between doctors and patients make it more complex to establish trust between doctors and patients online (70). This study conducted an in-depth analysis of the important factors that affect patients' online and offline trust, as well as the mechanism of trust transfer. The main conclusions are as follows:

Firstly, online healthcare provides patients with an unprecedented medical experience due to its convenience and immediacy. However, this virtual communication method also brings challenges in building trust (71). This study found that although there are many uncertainties in the online environment, the trust established by patients online can still significantly affect their trust in offline doctors, which reflects the practical significance of trust transfer theory. This discovery emphasizes the importance of online healthcare platforms in maintaining patient trust. In order to promote trust transfer, online medical platforms should strive to improve service quality, ensure accuracy and transparency of information, and strengthen professional training and certification of doctors to enhance patient confidence.

Secondly, the presentation of professional abilities in online healthcare differs from traditional offline healthcare. In the online environment, patients mainly perceive the professional abilities of doctors through their written descriptions, video consultations, and other means (72). Therefore, doctors need to pay more attention to improving their online communication skills and expression methods in order to effectively showcase their professional knowledge and experience in the online environment. Meanwhile, due to the time lag effect of establishing online trust on the perception of professional competence, online medical platforms can consider assisting patients in forming a comprehensive understanding of doctors' professional competence through patient evaluation, professional certification, and other methods.

Thirdly, service attitude also plays a crucial role in online healthcare. Online medical platforms should encourage doctors to demonstrate a positive and patient service attitude, and improve patient satisfaction and trust through instant replies, detailed answers, and other methods (73). In addition, online medical platforms can optimize service processes and improve service efficiency through technological means such as intelligent customer service and appointment reminders, thereby further enhancing patient trust.

Fourthly, treatment effectiveness, as a core element in building trust between doctors and patients, has shown significant impact in both online and offline healthcare. In order to improve the treatment effect, doctors need to continuously enhance their professional skills and medical level, while paying attention to communication and interaction with patients, understanding their needs and expectations. Online medical platforms can assist doctors in developing more personalized and accurate treatment plans by providing remote monitoring, data analysis, and other services, thereby improving treatment effectiveness and patient satisfaction (74).

In summary, there are significant differences between online healthcare and traditional offline healthcare in establishing trust between doctors and patients, but the two share commonalities in the core elements of trust building. This study provides useful suggestions for online medical platforms and doctors by analyzing the complex mechanisms of trust building between doctors and patients in both online and offline environments. In the future, with the continuous development and improvement of digital medical technology, online medical platforms should continue to explore how to better integrate online and offline resources, optimize medical service quality, enhance patient satisfaction, and establish a more stable doctor-patient trust relationship. At the same time, the government and regulatory agencies should strengthen supervision and guidance of online medical platforms to ensure the safety and effectiveness of online medical services, and provide patients with better quality medical services.

### 6.2 Theoretical contributions

Firstly, this study fills the gap in research on trust transfer mechanisms in the field of online healthcare by analyzing in depth the process of trust formation in patients' online medical platforms and how this trust is transferred to offline diagnosis and treatment activities. This discovery not only deepens our understanding of the dynamic evolution of trust, but also provides theoretical support for how online medical platforms can effectively promote trust transfer.

Secondly, this study not only focuses on a single path of trust transfer, but also comprehensively explores online and offline trust transfer from multiple dimensions, such as trust mechanisms, influencing factors, and conversion paths. This multidimensional analysis approach helps us to have a more comprehensive understanding of the complexity and diversity of trust transfer, providing new perspectives and methods for future research.

Furthermore, this study applies trust transfer theory to the emerging field of online healthcare platforms, which not only enriches the research content of trust in the medical field, but also provides new practical scenarios and theoretical support for the expansion of trust theory in the medical field.

### 6.3 Practical implications

The proposed integration model of online and offline services, online reputation management mechanism for doctors, and user feedback mechanism in this study provide specific practical guidance for online medical platforms on how to optimize service experience and enhance patient trust. These measures will help online medical platforms better meet the needs of patients and achieve seamless integration of online and offline medical services.

Specifically, we propose the following suggestions for policy makers:

Firstly, formulate policies for the integration of online and offline services. Promote the formulation of relevant policies, encourage the deep integration of online and offline medical services, and ensure that patients can enjoy continuous, consistent, and high-quality medical services across different service channels. Establish special funds or subsidies to support medical institutions in technological transformation and process optimization for the integration of online and offline services.

Secondly, establish an online reputation management system for doctors. Develop unified standards and methods for online reputation evaluation of doctors to ensure fairness and accuracy of the evaluation. Encourage third-party organizations to participate in the evaluation and certification of doctors' online reputation, and improve the transparency and credibility of reputation management.

Thirdly, improve the user feedback mechanism. Establish a sound mechanism for collecting, analyzing, and processing user feedback to ensure timely response and improvement of patient opinions. Making user satisfaction one of the important indicators for evaluating medical institutions and doctors, and linking it with medical insurance payments, professional title evaluations, etc.

Fourthly, strengthen Internet medical supervision. We will improve laws and regulations related to Internet medicine, clarify the responsibilities and obligations of all parties, and protect the rights and interests of patients. Strengthen the daily supervision and regular inspection of the Internet medical platform to ensure its compliance operation (75).

On the other hand, we offer the following suggestions to platform developers:

Firstly, optimize the online medical service experience. Utilize technologies such as big data and artificial intelligence to enhance the intelligence and convenience of online consultation, appointment registration, remote diagnosis and treatment services. Design a simple and intuitive user interface and operation process to lower the threshold for patients to use.

Secondly, strengthen the online reputation management function for doctors. Develop an online reputation display and evaluation system for doctors, allowing patients to evaluate and score doctors based on their real experiences. Introducing algorithms to dynamically update and rank the reputation of doctors, ensuring that high-quality doctors can stand out.

Thirdly, establish an efficient user feedback system. Develop multi-channel user feedback collection tools, including online questionnaires, customer service hotlines, social media, etc., to ensure the comprehensiveness and timeliness of feedback. Utilizing natural language processing and other technological means to intelligently analyze and classify user feedback, providing data support for improving services (76).

Fourthly, ensure data security and privacy protection. Strengthen data encryption and access control to ensure the security of patient personal information and medical data. Comply with relevant laws and regulations, clearly inform patients of the purpose and scope of data use, and obtain patient consent. The fifth is to promote technological innovation in the integration of online and offline services. Explore the use of technologies such as the Internet of Things and 5G to achieve seamless integration and collaboration of online and offline medical services. Develop intelligent medical devices that support remote monitoring, diagnosis, and treatment to improve the efficiency and accuracy of medical services (77).

In conclusion, this study reveals the key role of patient trust in the development of Internet medicine, and the importance of online and offline trust transfer mechanism. These findings will help policymakers and industry participants better understand and understand the development law of Internet medicine, so as to formulate more scientific and reasonable policies and management measures, and promote the healthy development of Internet medicine.

## 7 Research limitations and future prospects

Although we have made substantial progress in exploring the issue of patient trust on online medical platforms, revealing the phenomenon of patients transferring from online trust to offline trust in online medical platforms, we still face some research limitations, as follows:

(1) Method gap and model optimization

Although we employed text mining techniques for robust analysis, this study did not use qualitative methods such as patient interviews or focus groups to triangulate the results. This may affect the comprehensiveness and accuracy of our verification of trust transfer models. To make up for this deficiency, we plan to further conduct qualitative research in subsequent studies, through patient interviews and focus groups, to explore in depth the process of establishing and transferring patients' trust in online medical platforms, in order to more comprehensively validate and optimize our trust model.

(2) Contextual externalities and data acquisition

Due to limitations in data acquisition, this study may not fully reflect the changes and impacts of all relevant external factors. For example, certain policy shifts or technological adoption may have varying impacts in different regions or time periods. Future research can more accurately evaluate the effects of these external factors through broader data collection and analysis.

(3) Generalizability challenges and global perspective

The trust model proposed in this study may have limited applicability in different socio-cultural and regulatory environments. There are systemic differences in the global healthcare service system, including medical regulations, cultural backgrounds, patient behavior patterns, and other factors that may affect patients' trust building and transfer process toward online healthcare platforms. Therefore, we recognize that trust models may need to be adjusted and optimized accordingly in different environments. In order to meet this challenge, we plan to further investigate the impact of different social cultures and regulatory environments on trust models in future research, explore the trust transfer mechanism in the cross-cultural context, and how to adjust and optimize trust models in different environments to ensure their wide applicability.

In summary, by comprehensively considering issues such as method gaps, contextual externalities, generalization challenges, and deepening the understanding and strategy development of trust transfer mechanisms, we expect to achieve more comprehensive and in-depth results in future research, providing more effective support for trust building and patient trust transfer in online medical platforms. At the same time, we also look forward to collaborating with more researchers to jointly promote the development of this field.

## Data Availability

The original contributions presented in the study are included in the article/supplementary material, further inquiries can be directed to the corresponding author.
